# Circulating tumour cell liquid biopsy in selecting therapy for recurrent cutaneous melanoma with locoregional pelvic metastases: a pilot study

**DOI:** 10.1186/s13104-020-05021-5

**Published:** 2020-03-24

**Authors:** Stefano Guadagni, Giammaria Fiorentini, Ioannis Papasotiriou, Panagiotis Apostolou, Francesco Masedu, Donatella Sarti, Antonietta Rossella Farina, Andrew Reay Mackay, Marco Clementi

**Affiliations:** 1 Department of Applied Clinical Sciences and Biotechnology, University of L’Aquila, 67100 L’Aquila, Italy; 2 Department of Oncology and Hematology, Ospedali Riuniti Marche Nord, 61121 Pesaro, Italy; 3 Research Genetic Cancer Centre International GmbH, Zug, Switzerland; 4 Research Genetic Cancer Centre S.A, Florina, Greece

**Keywords:** Liquid biopsies, Circulating tumour cells, Precision oncotherapy, Recurrent melanoma, Hypoxic pelvic perfusion, Melphalan, Pelvic loco-regional metastases

## Abstract

**Objectives:**

Circulating tumour cells (CTCs) from liquid biopsies provide an exceptional opportunity to obtain real-time tumour information and are under current investigation in several cancers, including cutaneous melanoma, but face significant drawbacks in terms of non-standardised methodology, low viable cell numbers and accuracy of CTC identification. In this pilot study, we report that chemosensitivity assays using liquid biopsy-derived metastatic melanoma (MM) CTCs, from 7 patients with stage IIIC, *BRAF* wild-type metastatic melanomas, localized exclusively to the pelvic region, un-eligible for immunotherapy and treated with melphalan hypoxic pelvic perfusion (HPP), is both feasible and useful in predicting response to therapy. Viable MM CTCs (> 5 cells/ml for all 7 blood samples), enriched by transient culture, were characterised in flow cytometry-based Annexin V-PE assays for chemosensitivity to several drugs.

**Results:**

Using melphalan as a standard, chemosensitivity cut-off values of > 60% cell death, were predictive of patient RECIST 1.1 response to melphalan HPP therapy, associated with calculated 100% sensitivity, 66.67% specificity, 33.33% positive predictive, 100% negative predictive, and 71.43% accuracy values. We propose that the methodology in this study is both feasible and has potential value in predicting response to therapy, setting the stage for a larger study.

*Trial registration* Clinical Trials.gov Identifier NCT01920516; date of trial registration: August 6, 2013

## Introduction

Approximately 10% of cutaneous melanomas (CMs) recur as locoregional metastases following treatment [1]. Of these, ≈ 15% involve the pelvis [2], with only 2% of pelvic/inguinal metastases presenting in the absence of leg lesions [3]. Over the past 15 years, local, regional and systemic treatments for locoregional metastatic melanoma have evolved. For patients with stage III and IV CM with pelvic locoregional metastases, specialist melanoma centres provide local, regional, and systemic treatment options. Local treatments include surgical resection [4], topical therapy with diphencyprone or imiquimod [5], diathermy-fulguration, cryotherapy, laser ablation, radio-frequency ablation, intralesional injection with Rose Bengal [6] or Talimogene laherparepvec [7] or Darumon or Coxsackie Virus A-21, and electro-chemotherapy [8, 9]. Regional treatment options include regional radiation therapy [10, 11] and hypoxic pelvic perfusion (HPP), as isolated limb perfusion and isolated limb infusion are not anatomically practical for metastases targeting. HPP is achieved by blocking the blood circulation in the pelvic area at the aorta and inferior vena cava with balloon catheters and at thigh-level with pneumatic cuffs. The HPP rational, as for isolated limb infusion, is based upon the potential to expose locoregional melanoma metastases to higher drug concentrations, with the cytotoxicity of agents, such as melphalan, augmented by hypoxia [12]. We recently reported that melphalan HPP in 36 pre-treated patients with stage III and IV melanoma, with pelvic locoregional metastases in progression, resulted in an overall response rate of 94%, a median survival time of 15 months and a 5-years survival rate of 8% [3, 13–15]. Systemic therapies, either alone or in combination with local and/or regional therapies, are currently proposed when pelvic melanoma metastases are considered unresectable for technical and clinical reasons. In patients with unresectable stage III and IV, dabrafenib and trametinib *BRAF*/*MEK* inhibitors have resulted in 3-year overall survival (OS) rates of 44%, pembrolizumab, a monoclonal anti-PD-1 antibody, has resulted in 4-year OS rates of 44% and the combination of ipilimumab anti-CLTA-4 and nivolumab anti-PD-1 antibodies has resulted in three-year OS rates of 58%. However, in these studies, published over the past 5 years, unresectable stage III melanomas represent only 3% of cases, making the extrapolation of these data to patients with unresectable stage III melanoma localized to the pelvic region, challenging [16]. Furthermore, target therapy only provides a significant improvement in overall median survival for ≈ 50% of BRAF^V600E^ mutated patients [17, 18] and new immunotherapies are effective in ≈ 45% of *BRAF* wild-type patients [19–21]. Finally, patients with concomitant autoimmune disorders, chronic viral infections, organ dysfunction, organ transplant, brain metastases, or too old and frail, or pregnant, were in general excluded from immune checkpoint inhibitor clinical trials [22].

This apparent plethora of therapeutic options not only reflects the fact that not all treatments are available in each institution but also that no single strategy is suitable for every patient, with treatment choice dependent upon lesion number, size, anatomic location, the presence of regional lymph node or distant metastases and also biomolecular aspects, concomitant disease and previous therapy. For these reasons, we believe that treatment strategies for pelvic locoregional metastases should be multidisciplinary and could benefit greatly from the detailed characterisation of biomolecular characteristics and relative chemosensitivity of metastatic melanoma cells, a possibility that is offered by liquid biopsies, using purified circulating tumour cells (CTCs) obtained from individual patients. This method has been approved for prognosis by USA Food and Drug Administration [23] and despite a lack of methodological consensus, is under investigation as a potential method for identifying therapeutic strategies for cancers, including melanoma [24–26].

Here, we report a pilot study of CTCs purified from a homogeneous group of stage IIIC melanoma patients with locoregional, *BRAF* wild-type metastases located exclusively to pelvic region, who were not eligible for immune checkpoint inhibitor therapy and were submitted for melphalan HPP therapy. The aim of this study was to confirm both the feasibility and utility of assessing the chemosensitivity of CTCs purified from liquid biopsies, as a predictive test for selecting therapeutic strategy.

## Main text

This project was performed in accordance with the Declaration of Helsinki and was approved by the ethics committee of ASL n.1, Abruzzo, Italy (10/CE/2018, 19 July, n.1419). Written informed consent was obtained from each patient. From a prospective trial of melanoma patients undergoing melphalan perfusion/hypoxic infusion (Clinical Trials. gov Identifier NCT01920516), a subset of 41 were selected with stage III and IV patients with locoregional metastases located in the pelvis and/or inguinal region and/or upper third of the thighs. From this 41-patient cohort, 7 patients were selected with *BRAF* wild-type status, stage IIIC, not eligible for immunotherapy, submitted to melphalan HPP (Table 1A). Histopathological analysis revealed that all metastases exhibited an epithelioid phenotype. *BRAF* status and *MGMT* promoter methylation status were assessed as previously described [14]. Exclusion from immunotherapy was due to concomitant Hepatitis C infection in 4 patients, in treatment with sofosbuvir (400 mg) and daclatasvir (30 mg) and acute phase inflammatory bowel disease in 3 patients, treated with high dose corticosteroids. This study adheres to CONSORT guidelines.

**Table 1:** A—Clinical characteristics of the 7 stage IIIc melanoma patients with pelvic locoregional metastases. B—Liquid biopsy metastatic melanoma CTC chemosensitivity assays

Part A	Age (years)	Burden	≥ 1 mitosis/mm^2^	Chromotype	BRAF^V600E^ status	Percentage of *MGMT* promoter methylation	Previous therapies	Concomitant diseases -therapies	RECIST 1.1 response	Progression-free survival from 1st melphalan HPP -site of progression	Therapies at progression	Censor -Overall survival from 1st melphalan HPP			
Pt 1	50	High	Not	Black	Wild-type	10	Surgery Interferon alpha	Hepatitis C -Sofosbuvir and daclatasvir	SD	3 months -locoregional	2nd melphalan HPP	Dead -17 months			
Pt 2	55	Low	Yes	Black	Wild-type	14.2	Surgery Dacarbazine systemic chemotherapy	Inflammatory bowel disease -corticosteroids	SD	2 months -locoregional	2nd melphalan HPP; Platinum ECT	Dead -47 months			
Pt 3	60	High	Yes	Red	Wild-type	20.2	Surgery	Hepatitis C -Sofosbuvir and daclatasvir	PR	3 months -locoregional and distant	Dacarbazine systemic chemotherapy	Dead -19 months			
Pt 4	61	High	Yes	Black	Wild-type	12.2	Surgery	Hepatitis C -Sofosbuvir and daclatasvir	SD	3 months -locoregional and distant	Gemcitabine, paclitaxel systemic chemotherapy	Dead -7 months			
Pt 5	85	Low	Not	Black	Wild-type	21.2	Surgery Interferon alpha	Inflammatory bowel disease -corticosteroids	SD	3 months -locoregional	2nd melphalan HPP; surgery	Dead -21 months			
Pt 6	38	Low	Not	Red	Wild-type	16.2	Surgery Radiation therapy Dacarbazine systemic chemotherapy	Inflammatory bowel disease -corticosteroids Lymphoma NH B-CLL -corticosteroids	SD	4 months -locoregional	2nd melphalan HPP; surgery	Dead -21 months			
Pt 7	75	High	Not	Black	Wild-type	28.5	Surgery -Bleomycin ECT	Hepatitis C -Sofosbuvir and daclatasvir	SD	4 months -locoregional and distant	Dacarbazine systemic chemotherapy	Dead -15 months			

Part B	IV-CTCs	5-FU (%)	Gem (%)	Dacarb (%)	Epi (%)	Alk (%)	Eto (%)	Carbo (%)	Cis (%)	Ox (%)	Paclit (%)	Doce (%)	Vino (%)	Topo (%)	Iri (%)
Pt 1	9.6 ml, SD ± 0.3 cells	S = 30	S = 90	S = 80	S = 30	S = 60	S = 40	S = 45	S = 30	S = 30	S = 85	S = 58	S = 20	S = 20	S = 30
Pt 2	16.2/ml, SD ± 0.3 cells	S = 30	S = 85	S = 80	S = 25	S = 50	S = 50	S = 75	S = 90	S = 40	S = 50	S = 50	S = 50	S = 30	S = 30
Pt 3	6.4/ml, SD ± 0.3 cells	S = 20	S = 50	S = 70	S = 30	S = 65	S = 25	S = 30	S = 50	S = 30	S = 30	S = 40	S = 30	S = 25	S = 20
Pt 4	8.4/ml, SD ± 0.3 cells	S = 25	S = 85	S = 72	S = 25	S = 54	S = 24	S = 20	S = 30	S = 30	S = 90	S = 50	S = 30	S = 30	S = 30
Pt 5	9.8/ml, SD ± 0.3 cells	S = 35	S = 65	S = 30	S = 31	S = 72	S = 23	S = 30	S = 30	S = 30	S = 95	S = 60	S = 30	S = 30	S = 30
Pt 6	9.2/ml, SD ± 0.3 cells	S = 25	S = 30	S = 95	S = 25	S = 84	S = 90	S = 42	S = 35	S = 35	S = 20	S = 20	S = 25	S = 20	S = 20
Pt 7	9.8/ml, SD ± 0.3 cells	S = 50	S = 50	S = 75	S = 20	S = 50	S = 50	S = 50	S = 40	S = 50	S = 85	S = 30	S = 30	S = 20	S = 20

Low burden (< 10 nodules; or no lesion > 3 cm); High burden (≥ 10 nodules; or one lesion > 3 cm); *ECT* electrochemotherapy, *NH B-CLL* non-Hodgkin B lymphocytes, *PR* partial response, *SD* stable disease, *Pt* patient, *IV-CTCs* isolated viable circulating tumor cells, *5-FU* 5 fluorouracil, *Gem = gemcitabine*, *Epi* epirubicin, *Alk* melphalan, *Eto* etoposide, *Carbo* carboplatin, *Cis* cisplatin, *Ox* oxaliplatin, *Paclit* paclitaxel, *Doce* docetaxel, *Vino* vinorelbine, *Topo* topotecan, *Iri* irinotecan, *S* sensitivity

**Fig. 1:**
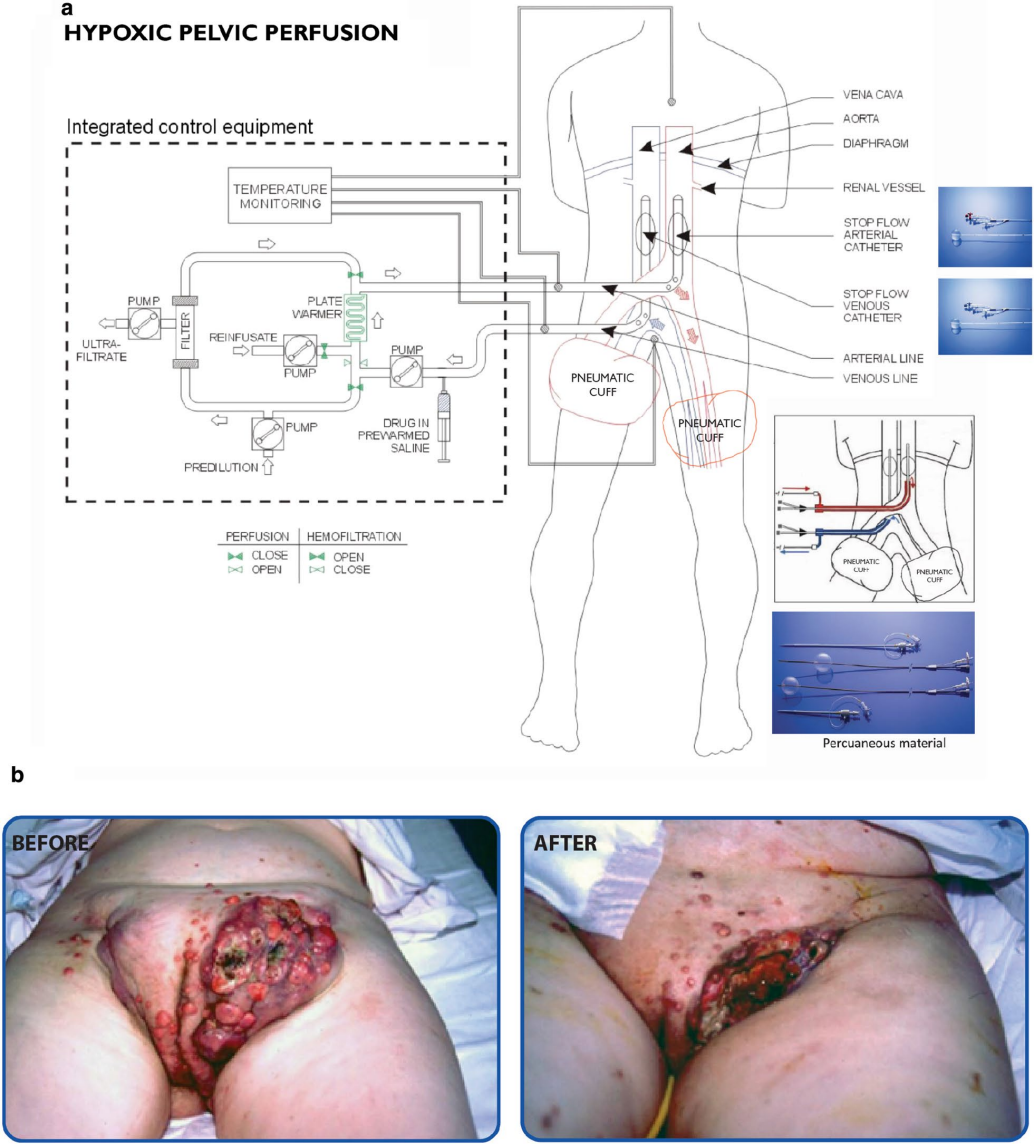
**a** Schematic representation of hypoxic pelvic perfusion (HPP) with chemofiltration (surgical and percutaneous procedures). **b** Stage IIIc female patient with pelvic melanoma locoregional metastases before and 2 weeks after HPP perfusion via the right iliac vessels

CTC purification and chemosensitivity assays are detailed in Additional file 1 and have been described previously [27, 28]. Briefly, metastatic melanoma CTCs were purified from blood samples by column-based magnetic cell separation, using CD45 magnetic beads. Purified CTCs were qRT-PCR validated for CD63 expression and assayed for sensitivity to chemotherapeutic agents in Annexin V-PE flow cytometry chemosensitivity assays. Surgical and percutaneous HPPs, followed by chemofiltration (Fig. 1a), were performed as previously described [13, 29]. Briefly, for HPP, pelvic circulation was isolated by blocking aortic and inferior vena cava blood flow with balloon catheters, introduced either percutaneously or surgically, and at thigh-level with pneumatic cuffs. Perfusion was performed under hypoxic conditions with low flow-rates (50–150 ml/min) and mild circuit hyperthermia in order to maintain tissue normothermia. Tumour response, according to Response Evaluation Criteria in Solid Tumors, version 1.1 [30], was assessed at ≈ 45 days following the 1st cycle of melphalan HPP. Responses for deep masses were evaluated by Computerized Tomography (CT), Magnetic Resonance Imaging (MRI) and Position-emission Tomography (PET) and superficial lesions were monitored by physical inspection with photographic comparison. Statistical analysis is descriptive due to the small sample size. Purified CTC numbers, progression-free survival (PFS) and OS times are presented as medians and interquartile range. The relationship between CTC melphalan chemosensitivity and disease response to melphalan HPP are presented, without confidence intervals, as percentages of sensitivity, specificity, positive predictive value (PPV), negative predictive value (NPV) and accuracy. All computations were performed using STATA statistical software.

More than 5 viable metastatic melanoma cells/ml were isolated in liquid biopsies of all 7 patients (Table 1B), median number 9.6/ml (interquartile range 8.4–9.8). The chemosensitivity of CTCs are presented in Table 1B. As concerning melphalan, the drug used for HPP, a chemosensitivity cut-off value > 60% cell-death was observed in CTCs from 3 patients. RECIST 1.1 responses to melphalan HPP for the 7 patients are presented in Table 1A. A partial response (PR) occurred in only 1 patient (Fig. 1b). Positive (complete or partial) and negative (stable disease or progression) RECIST 1.1 responses, following melphalan HPP, associated with CTC melphalan chemosensitivity > 60% or < 60%, in terms of dead/dying cells, are displayed in Table 2. A 100% sensitivity value was observed for complete or partial RECIST 1.1 response, following melphalan HPP, associated with > 60% CTC chemosensitivity to melphalan and a 66.67% specificity value was observed for stable or progression RECIST 1.1 disease responses, following melphalan HPP, associated with < 60% CTC chemosensitivity to melphalan. A 33.33% PPV for a positive RECIST 1.1 response was associated with > 60% CTC chemosensitivity to melphalan and a 100% NPV for a negative RECIST 1.1 response was associated with < 60% CTC chemosensitivity to melphalan. The overall capacity of CTC chemosensitivity tests to predict response to melphalan HPP (accuracy value) of 71.43% was calculated from the ratio of positively and negatively corrected classified patients, using a melphalan chemosensitivity cut-off value of 60% and RECIST 1.1 criteria, and the total number of patients treated with melphalan HPP.

**Table 2:** Positive (complete or partial) and negative (stable disease or progression) RECIST 1.1 responses after melphalan HPP, associated with > 60% (Positive) or < 60% (Negative) melphalan-treated CTC cell death

Chemosensitivity of CTCs	RECIST 1.1 response		Total
	Positive (CR + PR)	Negative (SD + PD)	
Positive (> 60%)	1	2	3
Negative (≤ 60%)	0	4	4
Total	1	6	7

*CR* complete response, *PR* partial response, *SD* stable disease, *PD* progressive disease

PFS (Table 1A) ranged from 2 to 4 months (median 3 months, interquartile range 3–4 months). Locoregional progression occurred in 3 patients, with locoregional plus distant site progression observed in the other 4 patients. In accordance with CTC chemosensitivity tests, each patient received chemotherapeutic agents based upon > 60% CTC chemosensitivity to that agent. Based on CTC chemosensitivity suggestions, 4 patients with locoregional plus distant metastases received systemic chemotherapy only and 3 patients with locoregional relapse received locoregional treatments (Table 1A). All patients subsequently died from disease progression, associated with OS times ranging from 7 to 47 months (median 19 months, interquartile ranges 15–21 months).

This pilot study was initiated to assess the feasibility of using purified CTCs, in terms of reproducibility, sampling, storage, transport, purification and enrichment methodologies, and the utility and suitability of subsequent CTC chemosensitivity assays in selecting therapeutic strategies and predicting response. For this purpose, we selected a homogeneous group of 7 stage IIIC melanoma patients with *BRAF* wild-type status and locoregional metastases located exclusively to the pelvic region, all of whom were un-eligible for novel immunotherapy and were treated with melphalan HPP, in accordance with percentage *MGMT* promoter methylation levels in tissue-specimen, as a relevant index of melphalan efficacy [14].

The numbers of metastatic melanoma CTCs from all liquid biopsies were greater that the cut-off value of 5 CTCs/ml, required for chemosensitivity assays. The interval between blood sampling and qRT-PCR analysis did not exceed 80 h, which has previously been reported to minimise gene and protein expression alterations [27]. We also report a 100% detection rate for patient CTCs, which is higher than previous reports of CTCs purified from liquid biopsies from primary cancers patients [31], which can be explained by increased numbers of CTC in high tumour burden stage IIIc metastatic melanomas. We demonstrate that the Annexin V-PE flow cytometry methodology employed for in vitro chemosensitivity assays, provided useful information concerning the CTC sensitivity to a variety of chemotherapeutic agents. In contrast to tissue validated chemosensitivity assays, CTC chemosensitivity assays do not preserve cell-to-cell or cell-to-matrix interactions [32]. For this reason, a cut-off value of > 60% was chosen for CTC chemosensitivity analyses, which is higher than > 30% in tissue chemosensitivity tests that may better resembling tumour structure [32].

In order to evaluate the predictive potential of in vitro CTC chemosensitivity assays in prognosis and therapeutic strategy, melphalan was employed as the principle drug, with patient RECIST responses to melphalan HPP used to evaluate sensitivity, specificity, accuracy and predictive potential. This resulted in values of 100% for sensitivity, 66.67% for specificity, 33.33% for PPV, 100% for PNV and 71.43% for accuracy, which were better overall values than those of 85.7% for sensitivity, 18.2% for specificity, 40% for PPV, 66.7% for PNV and 44.44% for accuracy, reported for in vitro tissue-validated chemosensitivity assays in ovarian cancers, applying a cut-off value of 30% [32]. Our observations are also in line with predictive potential of percentage *MGMT* promoter methylation, which was assessed in tissue biopsies from all 7 patients and was also predictive for melphalan efficacy [14].

This pilot study supports the feasibility of a methodology for liquid biopsy sampling, storage, transport, CTC purification, transient in vitro CTC culture and subsequent use of CTCs in chemosensitivity assays. We demonstrated: (i) the numbers of CTCs purified from liquid biopsies obtained from melanoma patients with locoregional metastases, is sufficient for Annexin V-PE flow cytometry-based chemosensitivity tests; (ii) in vitro CTC chemosensitivity assays are feasible to predict RECIST 1.1 responses to locoregional melphalan chemotherapy with an accuracy value of 71.43%.

## Limitations

The scope of this pilot study was to provide preliminary evaluation of the feasibility of this CTC-based methodological approach, as a forerunner to a larger cohort study, depending upon results, and was not designed to evaluate treatment safety, efficacy or effectiveness [33], justifying the small sample size analysed and absence of inferential statistical analysis. Stringent recruitment parameters, such as sample homogeneity, furthermore mitigate small sample sizes, and are pre-requisites for determining feasibility [33]. We caution that the feasibility results obtained in this pilot study, quantified as percentage sensitivity, specificity, PPV, PNV and accuracy, are at most indicative and should not be extrapolated to patient inclusion and exclusion criteria not used in this study. Furthermore, the use of CTC chemosensitivity assays, such as in this report, should not yet be considered to represent a validated preliminary test for therapeutic selecting, for two fundamental reasons: (i) the current lack of accepted and standardised methodologies for CTC isolation, purification, enrichment, characterisation and use in chemosensitivity assays, and (ii) the small sample size analysed. We stress that the efficacy data presented are uncontrolled and observational.

## Supplementary information

**Additional file 1.**Methodology for liquid biopsies, CTCs chemosensitivity assays, and molecular evaluation.

## Data Availability

All data generated or analysed during this study are included in this published article and are available from the corresponding author on reasonable request.

## Abbreviations

CMs: Cutaneous melanomas
HPP: Hypoxic pelvic perfusion
OS: Overall survival
MGMT: O^6^-methylguanine-DNA methyltransferase
BRAF: v-Raf murine sarcoma viral oncogene homolog B
MEK: Mitogen-activated protein kinase–kinase enzymes
PD-1: Programmed cell death protein 1
CLTA-4: Cytotoxic T-lymphocyte-associated protein 4
CTCs: Circulating tumor cells
USA: United States of America
RECIST: Response evaluation criteria in solid tumors
qRT-PCR: Quantitative reverse transcription polymerase chain reaction
PPV: Predictive positive value
PNV: Predictive negative value
FFPE: Formalin fixed paraffin embedded
CAST: Competitive allele specific technology
PCR: Polymerase chain reaction
MS-MLPA: Methylation-Specific Multiplex Ligation-Dependent Probe Amplification
Hha1: Calpastatin Hha1 gene
EDTA: Ethylenediaminetetraacetic acid
CD45: Protein tyrosine phosphatase, receptor type, C
CD63: Protein encoded by the *CD63* gene
FBS: Fetal bovine serum
RPMI-1640: Growth medium used in cell culture
CD31: Platelet endothelial cell adhesion molecule
CT: Computerized tomography
MRI: Magnetic resonance imaging
PET: Positron-emission tomography
SD: Standard deviation
PFS: Progression free survival
