# Supplementary material for: Circulating tumour cell liquid biopsy in selecting therapy for recurrent cutaneous melanoma with locoregional pelvic metastases: a pilot study
Source: BMC Res Notes. 2020 Mar 24;13:176. doi: 10.1186/s13104-020-05021-5 (PMC7092420; doi:10.1186/s13104-020-05021-5)
Supplement: Supplementary file 1 — Additional file 1.Methodology for liquid biopsies, CTCs chemosensitivity assays, and molecular evaluation. [file 13104_2020_5021_MOESM1_ESM.docx]

*Liquid biopsies and CTCs chemosensitivity assays*

For CTCs, 20 ml of blood, containing 7 ml of 0.02 M EDTA, were collected from each patient in sterile 50 ml Falcon tubes and stored at 2-8 °C. Stored tubes were placed in impact-resistant transportation containers, transported under refrigeration and analysed within 80 hours. For CTCs purification, blood samples were layered over 4 ml polysucrose solution (Biocoll separating solution 1077, Biochrom, Berlin, Germany) and centrifuged for 20 min at 2500×g. Mononuclear cells, lymphocytes, platelets and granulocytes were collected and washed with phosphate-buffered saline (PBS, P3813; Sigma-Aldrich, Germany), incubated for 10 min in lysis buffer [154 mM NH_4_Cl (31107; Sigma-Aldrich), 10 mM KHCO_3_ (4854; Merck, Germany) and 0.1 mM EDTA in deionized water] to lyse erythrocytes, centrifuged, re-washed in PBS then incubated with CD45-conjugated magnetic beads (39-CD45-250; Gentaur, Belgium) and CD63 magnetic beads (39-CD63-250; Gentaur) for 30 min at 4°C. Following incubation, cells were collected in a magnetic field, washed in PBS and purified CD63 positive/CD45-negative cells cultured in 12-well plates (4430400N; Orange Scientific) in RPMI-1640 plus 10% FBS for chemosensitivity, viability and qRT-PCR assays. Purified peripheral blood mononuclear cells (PBMCs) from each patient were used as non-cancer cell controls. CTCs were validated by qRT-PCR, using specific primers for CD63 and other cell types excluded, using primers for CD31 and N-cadherin. Samples for chemosensitivity and gene expression assays contained ≥ 5 viable CTCs/ml.

For chemosensitivity assays, CTCs were cultured in 12-well plates (3513, Corning) and treated with the following drug concentrations: 10 μM melphalan (Μ2011, Sigma-Aldrich), 1 μM cisplatin (P4394, Sigma-Aldrich), 10 μM 5-fluorouracil (F6627, Sigma-Aldrich), 1,12 μM oxaliplatin (O9512, Sigma-Aldrich), 1 μM carboplatin (41575-94-4, Sigma-Aldrich), 5 μM irinotecan (I1406, Sigma-Aldrich), 500μM dacarbazine (D2390, Sigma-Aldrich), 10 nM paclitaxel (T7402, Sigma-Aldrich), 10nM docetaxel (01885, Fluka), 5 μΜ etoposide (E1383, Sigma-Aldrich), 50 nM vinorelbine (V2264, Sigma-Aldrich), 0.5 μM topotecan (T2705, Sigma-Aldrich), 50 nM gemcitabine (G6423, Sigma-Aldrich) and cell viability assessed by flow cytometry (BD Instruments Inc., San José, CA), using Annexin V-PE (559763; BD Bioscience), at 24-hour intervals for 6 days and the percentage of living, dead and dying apoptotic cells evaluated, using BD CellQuest Software (BD Instruments Inc). Chemosensitivity and viability validation was also corroborated using methyl-tetrazolium dye (MTT), crystal violet dye (CVE) and Sulfo-Rodhamine B (SRB) assays. The percentage of non-viable cancer cells was calculated under non-drug and drug-treated conditions, and chemosensitivity classified as: 1) non-sensitive <35%; 2) partially sensitive 35%-80%, and 3) highly sensitive >80%.

*Molecular Evaluation*

DNA was purified from 5x10 μm formalin-fixed paraffin-embedded tissue sections, using the DNA mini kit as directed by the manufacturer (Qiagen, Hilden, Germany) and DNA concentrations and quality determined in a Qubit fluorometer (Thermo-Fisher, Foster City, California, USA) and *BRAF* status determined using competitive allele-specific hydrolysis probes (TaqMan) and PCR technology (CAST) (Thermo-Fischer Scientific, Waltham, MA, USA).

*MGMT* promoter methylation status was assessed using an MS-MLPA probe mix (MRC-Holland, Amsterdam, Netherlands) (Salsa MS-MLPA Kit ME011 MMR), containing 6 *MGMT* promoter-specific probes for a region containing an *Hha1* recognition site, and the procedure performed according to the manufacturer’s directions and reactions incubated with *Hha1* restriction enzyme (R6441; Promega, Madison, Wisconsin, USA), which cuts unmethylated but not methylated GCGC sites. Resulting fragments were separated by capillary gel electrophoresis in a 3500 Genetic-Analyzer (Thermo-Fisher). CpGenome universal methylated DNA and unmethylated DNA (Chemicon; Millipore, Billerica, Massachussetts, USA) standards were included as controls in each MS-MLPA set and *MGMT* promoter methylation status subsequently quantified, using GeneMarker software (version 1.5; Soft Genetics, State College, Pennsylvania, USA). To compensate for the differences in PCR efficiency in individual samples, peak values for each probe were normalized by dividing test and control probe peak values. The *MGMT* promoter methylation status and methylation ratio was calculated by dividing mean normalized peak values for digested samples by the corresponding mean peak values for undigested samples and presented the percentage of methylated sequences.
